# The Swedish father/non-birthing parent visit: evaluating implementation fidelity among child health nurses one year after voluntary implementation

**DOI:** 10.1186/s12912-022-01011-z

**Published:** 2022-08-16

**Authors:** Rahel-Ochido Ibilola Odonde, Olov Aronson, Michael B. Wells

**Affiliations:** 1 St. George’s University Hospitals NHS Foundation Trust, Blackshaw Road, London, SW17 0QT UK; 2 School of Humanities, Education and Social Sciences, Örebro University, Örebro, Sweden; 3 Department of Women’s and Children’s Health, Karolinska Institutet, Solna, Sweden

**Keywords:** Child health nurses, Father visits, Cross-sectional, Sweden, Evaluation, Community-based intervention, Adherence, Implementation fidelity

## Abstract

Swedish child health centres (CHCs) have created a series of visits for fathers/non-birthing parents. The primary aim was to assess child health nurses’ implementation fidelity of the father/non-birthing parent visits, with a secondary aim of exploring predictor variables for fidelity. In 2017, nurses voluntarily implemented a series of father/non-birthing parent visits in Region Stockholm. Nurses (*n* = 122) completed baseline and 8—12 month follow-up surveys. Multiple imputation was used for missing data. Register data on the number of fathers attending the three-to-five month visit was used. Frequencies of nurses reporting good overall adherence to the home visit, three-to-five week visit, and three-to-five month visit were 86%, 76%, and 68%, respectively. A total of 3,609 fathers attended the three-to-five month visit in 2018, where over half of the visits were at 14 of the 134 CHCs. Multiple linear regression showed that working for a private CHC, seeing more fathers, and nurses’ perceptions of receiving enough support predicted higher three-to-five month visit adherence. After nurses saw eight fathers, they were more likely to adhere to the guidelines.

**Supplementary Information:**

The online version contains supplementary material available at 10.1186/s12912-022-01011-z.

## Introduction

Fathers’ mental health and wellbeing during the transition to fatherhood remains relatively unsupported [1–3]. Fathers are expected to be more involved as caregivers to their children compared to previous generations [4, 5], and they desire an increased share of responsibility in their children’s lives [6, 7]. To aid them in their transition, fathers expect and rely on support from [4] and a relationship with [8, 9] paediatric professionals, such as child health nurses. However, research suggests that mothers receive more professional support than fathers from child health professionals [4]. Consequently, fathers feel disregarded and excluded by child health professionals [8, 10, 11], which can lead them to not being involved early on in their child’s life, and a higher probability that they will not remain involved in their child’s health care [8, 10]. This in turn affects fathers’ parenting abilities [12], with the added result of placing more responsibility on mothers to manage their child’s health. Therefore, developing efforts to professionally support fathers may benefit the child, the father, and the whole family.

### Swedish child health centres

The Swedish healthcare system prioritises prevention [13] and therefore has shifted from a treatment-orientated model to a preventative one within the child healthcare services [11]. Child health nurses run the child health centres (CHCs) and see 99% of children in Sweden [11, 13]. CHCs’ support comes in many guises from providing preventative health care, such as vaccinations, assessing child development and treating morbidities that may develop throughout early childhood (0 – 5 years of age), as well as helping parents develop their parenting skills [11, 14].

While CHC policies are explicit in stating that both parents are important for everyone in the family [15], they have not supported both parents to the same degree [5, 11, 16]. However, Swedish nurses’ attitudes toward seeing fathers as equal parents has increased throughout the 21^st^ Century. For example, in 2004, around one-third to one-half of Swedish child health nurses were ambivalent toward fathers as carers of infants [9]. In 2014, nurses’ attitudes were more gender equal, with only around one-quarter to one-third of nurses being ambivalent toward fathers [16]. Despite their positive attitude changes, nurses note that it can be difficult to engage fathers, and no specific interventions have been initiated toward empowering nurses to better support fathers [11].

To better engage fathers, there should be specific supports aimed at them and their transition to parenthood [17–19]. Between 2013 and 2015, Region Stockholm introduced a pilot study consisting of a series of postnatal father/non-birthing parent visits and found that both nurses and fathers appreciated these visits [14]. In 2017, the region decided to start implementing a series of three father visits: i) a home visit, typically during the first week after birth, ii) a three-to-five week visit, and iii) a three-to-five month visit. The first two visits were already part of routine care, but now both parents should be explicitly invited to attend and participate. The three-to-five month visit is completely new and is only attended by the father/non-birthing parent and the infant. However, in 2017, the father/non-birthing parent visits were carried out by nurses voluntarily; that is, the visit would not be a mandatory part of the Swedish child health program, but any CHC wishing to include the visits as part of their care was permitted to do so. Region Stockholm is the most populous region in Sweden [15] and has the potential to reach and support approximately 25% of families in Sweden [14, 20]. In 2017, there were 483 nurses employed in this region, and each fully-employed nurse saw around 69 infants per year [20].

New interventions need to be evaluated for their effectiveness and outcomes [21]. Part of this assessment is determining whether the new father/non-birthing parent visits have been implemented as initially designed, known as implementation fidelity [22–24]. Understanding fidelity is important, as it helps determine whether or not the outcomes are due to the study design [22–24], offering some reasoning as to why this intervention succeeds, or conversely, needs adaptation [24].

The primary aim was to quantitatively assess child health nurses’ self-reported implementation fidelity of the father/non-birthing parent visits. A second aim was to explore which variables predicted higher implementation fidelity among nurses. A third aim was to see if there was a cut-off regarding the number of three-to-five month visits nurses had to complete before reporting higher adherence scores. A final aim was to describe how much more support nurses needed to best support all types of fathers.

## Methods

The current study uses Carroll et al.’s [24] evaluation of implementation fidelity, also known as adherence, as a means of evaluating the nurses’ implementation of the new father/non-birthing parent visits. Carroll et al. [24], proposed a conceptual framework, where adherence is recognised as the underpinning of implementation fidelity. In this conceptual framework, adherence is the combination of four subcategories: (i) content, (ii) coverage, (iii) frequency and (iv) duration [24]. Adherence to an intervention and the quality of service delivery have been shown to be linked [25].

All CHCs in Region Stockholm were invited to participate in the voluntary intervention. In 2017, a total of 314 nurses volunteered to attend a half-day training on implementing the new clinical father/non-birthing parent visits. All nurses who participated in the half-day training session were recruited for this study.

### Nurses’ training

Nurses could attend one of three half-day training sessions in 2017 (May, September, or December). The half-day training involved seminars that covered topics such as the importance of fathers, areas that fathers may require further support in, including bonding with their infants, co-parenting, parental leave usage, and screening for postnatal depression. There was also formal training on documentation of data gathered during visits. Of note, the client medical record software only allowed for the documentation of male partners; as only male personal identity numbers (personnumers) were accepted in the computer system. Therefore, it was not possible to know whether parents in female same-sex relationships (or other genders) attended visits. Lastly, all attending nurses were informed of how the implementation would be evaluated. Before the training ended, participants were handed implementation manuals and were notified that they could receive up to two one-on-one mentoring sessions to further ensure implementation fidelity. Nurses were then informed that they could immediately start the father/non-birthing parent visits.

### Study design

The current study is part of a larger study known as the Pappor/Icke-Födande Föräldrar study (PIFF; Fathers/Non-birthing Parents Study). All nurses who attended the half-day training could complete a survey on their socio-demographic background. These nurses were then emailed a link to an online adherence survey at eight to twelve months post-training (most nurses completed the survey around 11 months). Of the 314 nurses who attended the half-day training, 293 (93.3%) completed the background questionnaire, and 248 (79.0%) were at a CHC that saw at least one father in 2018. Of those nurses, 122 completed both the background and the adherence surveys (49.2% response rate), representing 55 CHCs. The 122 nurses who completed both surveys comprised the study sample. Table 1 includes a comparison of the organisational affiliations, educations, and experience of the 122 nurses in the study sample with the equivalent statistics for all 293 nurses who completed the background questionnaire. The comparison indicates that there were no significant differences between the study sample and all nurses who completed the background questionnaire, suggesting that the study sample seemed to reflect the total group of nurses who participated in the training session.

**Table 1:** Descriptive statistics

	Study sample (*n* = 122)				Completed background survey (*n* = 293)				
	Freq	%	M	SD	Freq	%	M	SD	*p*-value
Organization:									0.100 ^a^
County council	46	42			137	51			
Private	64	58			131	49			
Education:									0.448 ^a^
Pediatric nurse	56	51			143	55			
District nurse	54	49			116	45			
Experience (years):									0.317 ^b^
≤ 5	47	42			133	49			
6—10	29	26			60	22			
11- 15	13	12			31	11			
16—20	8	7			23	9			
≥ 21	14	13			25	9			
Visits per nurse			13.90	10.42			.	.	
I get enough support, to support the fathers			5.70	1.45			.	.	

*Note*: ^a^Based on chi-square test comparing study sample and all who completed background survey

^b^Based on Mann–Whitney U test comparing study sample and all who completed background survey

The socio-demographic data that were collected during the half-day training were merged with the participants’ adherence data. The survey was linked to their e-mail, and respondents received three reminder e-mails to complete the survey. The current study assessed their self-evaluated implementation fidelity when delivering i) the home visit, ii) the three-to-five week visit, and iii) the three-to-five month visit.

## Measures

### Nurses’ background information

Data taken during the half-day training, focused on the nurses’ background and were collected via a paper survey immediately before the half-day training. The child health nurses answered four items that related to their background: i) age, with response options: < 30, 30–39, 40–49, 50–59, ≥ 60; (ii) years of experience as a child health nurse, with response options: ≤ 5 years, 6–10, 11–15, 16–20, > 20 years; (iii) their nursing education (paediatric or district nurse); and (iv) the organisation they worked for (public = 1; private = 0). Nurses also reported their name and the name of their CHC so that their data could be linked with future surveys.

### Nurses’ self-reported adherence to the guidelines for the three father/non-birthing parent visits

This survey included nurses self-evaluating their adherence to the guidelines during three visits: (i) the home visit (7 items), (ii) the three-to-five week visit (10 items), and (iii) three-to-five month visit (13 items). Each item was answered on a Likert scale with seven response options, where 0 = completely disagree and 6 = completely agree. A scale was computed for each visit by summing the included items (see Supplementary Table). The scales were understood as formative rather than reflective, and they were not assumed to be unidimensional. Therefore, it was not relevant to perform tests of internal consistency.

### Nurses need of support to encourage fathers to attend

One item generically asked if nurses needed more support: I received enough support to support fathers. This item was asked on a Likert scale from 1 = completely disagree to 7 = completely agree. Nurses were then asked four additional items regarding the extent to which they needed more support to encourage fathers from different backgrounds to attend the visits: (i) Swedish-born fathers, (ii) fathers with a low-income, (iii) foreign-born fathers, and (iv) non-Swedish speaking fathers. The items were answered on Likert scales with seven response options, where 1 = least support needed and 7 = most support needed.

### Registered father/non-birthing parent visits

Data on the number of fathers/non-birthing parents attending the three-to-five month visit at each CHC in 2018 were used as predictor variables of nurses’ implementation fidelity. The register data were gathered from Region Stockholm’s Healthcare administration (Hälso- och sjukvårdsförvaltningen) system. Data from 2018 was collected in late January 2019 to ensure all 2018 visits had been registered. In total, there were 3,609 registered three-to-five month visits in the region.

Since the registered data were only reported on the CHC level, we took the total number of father/non-birthing parent visits divided by the number of nurses working at each CHC to calculate an average number of visits per nurse.

### Analysis

The patterns of missing data were assessed through the package finalfit in R [26], and in several instances, the results were not missing completely at random (MCAR). For this reason, multiple imputations of data were performed and pooled through the multivariate imputation by chained equations (MICE) package in R [27]. Predictor variables were drawn from the entire material and selected based on correlations with the quickpred function in MICE. The imputations created 100 complete datasets through 20 iterations, the convergence of which was checked with trace plots.

The proportions of nurses that adhered to the guidelines to an acceptable extent were estimated. The estimation of proportions was based on the original unimputed dataset because there did not appear to exist any accepted conventions for computing pooled confidence intervals for binomial proportions based on multiple imputed data. A cut-off for the summed adherence to each scale was created, where nurses who on average reported at least four out of six on the Likert-scaled items were categorised as adhering to the guidelines to an acceptable extent. The cut-off for the home-visit scale (with 7 items) was set to 28, the cut-off for the three-to-five week visit scale (with 10 items) was set to 40, and the cut-off for the three-to-five month visit scale (with 13 items) was set to 52.

Multiple linear regression models were estimated and pooled based on the imputed data sets. Control variables were selected to control for work and organisational conditions, while avoiding multicollinearity. It was not possible to include sex as a control variable, because nearly all nurses were female. Also, it was not possible to include age as a control variable since this variable had a correlation of *r* = 0.71 with experience as a nurse.

Comparisons were made between how much additional support nurses needed to encourage attendance from Swedish-born fathers, fathers with a low-income, foreign-born fathers, and non-Swedish speaking fathers. These comparisons were based on the imputed data sets. First, a repeated-measures ANOVA was performed, because the same group of nurses responded to each of the items. Since the ANOVA was significant (see the Results section), pairwise t-tests were made. The t-tests compared support needed to invite Swedish-born fathers with the items for each of the other groups of fathers.

Lastly, we examined if there was a cut-off for the association between the average number of father visits per nurse at each CHC and the nurses’ adherence to the guidelines for the three-to-five month visit. Since we do not have register data for the home visit or the three-to-five week visit, only the three-to-five month visit was assessed. The average number of registered father/non-birthing parent visits per nurse was re-categorised into one of four groups: seven or fewer visits (*n* = 32), 8 to 16 (*n* = 30), 16 to 23 (*n* = 22), and 24 or more (*n* = 22) visits. An independent samples t-test was performed to compare the adherence of the nurses who worked at CHCs with less than eight father visits per nurse to the adherence of those nurses who worked at CHCs with more visits. In an illustrative graph, the mean values for adherence were positioned at the centre of each bar, and the extension of each bar represented the interval between + 1 and -1 standard error from the mean.

### Ethical considerations

Formal ethical approval was not required in Sweden since the nurses’ data only referred to normal occupational responsibilities. However, all ethical considerations still followed the Declaration of Helsinki, as well as the European Union General Data Protection Regulation 2016/679 (EU GDPR). All nurses were informed verbally and in writing, via a survey received at their half-day training sessions and again in an online survey, regarding their participation in this research study prior to obtaining their informed consent. They were made aware that participation was voluntary and they could withdraw at any time, for any reason, without penalty for not participating. All data were anonymised prior to analysis and reporting.

## Results

In 2018, the first full year post-training, nurses from Region Stockholm registered 3,609 father visits at 80 of the 134 CHCs. Of these, 14 CHCs registered 52% of all three-to-five month visits (averaging 133 three-to-five month visits per CHC), while 24 CHCs registered between one to nine visits for the whole year. Of those CHCs who saw one or more fathers in 2018, CHCs, on average, registered 45.1 three-to-five month visits.

Considering adherence, 86% of participants self-reported that they adhered to the guidelines for the home visit (95% CI [0.78, 0.92]), 76% reported that they adhered to the guidelines for the three-to-five week visit (95% CI [0.66, 0.84]), and 68% reported that they adhered to the guidelines for the three-to-five month visit (95% CI [0.58, 0.77]). Nurses’ background factors are presented in Table 1. Over half of the nurses worked in a private CHC, and around half were paediatric nurses. When considering nurses’ experience levels, nurses working five or fewer years comprised the largest group. Overall, nurses felt mostly confident that they did not require more support to generally support fathers.

Table 2 presents three regression models with three different independent variables: adherence to the guidelines at the home visit, at the three-to-five week visit, and at the three-to-five month visit, respectively. According to the table, the nurses adhered to the guidelines for the home visit to a *greater* extent if they had more experience (b = 0.86, *p* = 0.009) and if they reported that they received enough support to support fathers (b = 0.86, *p* = 0.005). Likewise, nurses followed the guidelines for the three-to-five week visit to a *greater* extent if they reported that they received enough support to support fathers (b = 1.62, *p* = 0.011). Nurses followed the guidelines for the three-to-five month visit to a *lesser* extent if they worked for the county council (b = -6.83, *p* = 0.011), and they followed the guidelines to a *greater* extent if they worked at a CHC that received more fathers per nurse (b = 0.36, *p* = 0.003) and if they received enough support to support fathers (b = 3.59, *p* < 0.001).

**Table 2:** Multiple linear regression model with adherence at the home visit, at the three-five week visit, and at the three-five month visit as the dependent variables (*n* = 121)

	Home visit				3–5 week visit				3–5 month visit			
		95% CI				95% CI				95% CI		
	b	From	To	p	b	From	To	p	b	From	To	p
Organization: county council	0.09	-1.81	1.98	0.927	-1.91	-5.73	1.90	0.323	-6.83	-12.06	-1.60	0.011*
Visits per nurse	0.00	-0.08	0.09	0.914	-0.08	-0.25	0.09	0.342	0.36	0.13	0.59	0.003**
Education: pediatric	0.42	-1.45	2.29	0.654	-0.12	-3.88	3.64	0.950	-0.72	-5.84	4.40	0.781
Experience	0.86	0.21	1.50	0.009**	0.28	-1.03	1.59	0.674	-0.33	-2.11	1.45	0.713
I get enough support, to support the fathers	0.86	0.26	1.47	0.005**	1.62	0.39	2.86	0.011*	3.59	1.91	5.26	< 0.001***
R^2^	0.13				0.07				0.26			

^*^p < 0.05, **p < 0.01, ***p < 0.001

**Fig. 1:**
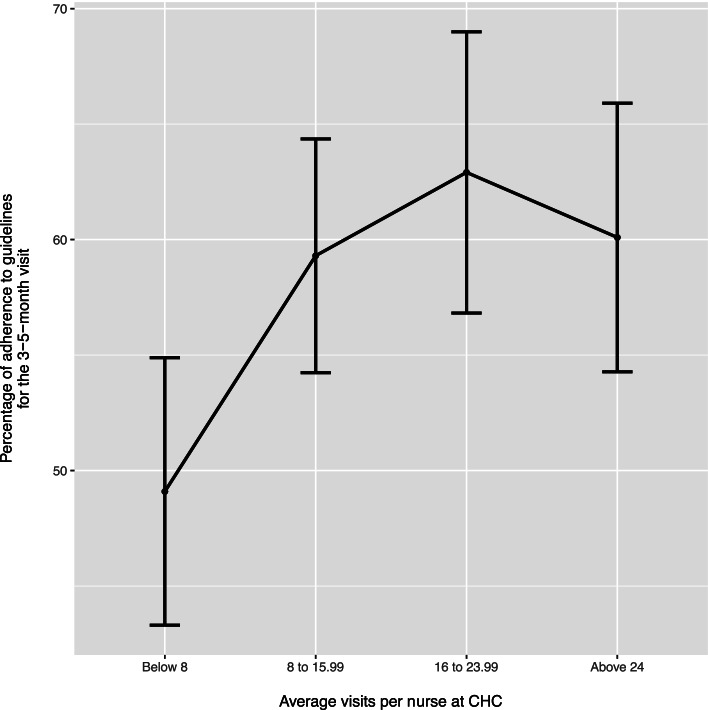
Graph representing the percentage of adherence to the guidelines for the 3–5-month visit by nurses working at CHCs with the averaged visit per nurse at that CHC. The bars indicate standard errors

Figure 1 includes a graph that represents the association between the average number of fathers received at each CHC and adherence to the guidelines for the three-to-five month visit. The graph indicates that nurses adhered less to the guidelines when they worked at CHCs that received, on average, less than eight fathers per nurse. An independent-samples t-test indicated that nurses who worked at CHCs that received eight or more fathers per nurse adhered to the instructions for the three-to-five month visit to a significantly greater extent than nurses who worked at CHCs that received less than eight fathers per nurse (*p* < 0.001).

Table 3 presents the means for the support nurses reported that they needed to encourage different groups of fathers to attend the visits. Nurses reported that they needed the least support to encourage Swedish-born fathers and the most support to encourage non-Swedish speaking fathers to attend visits. Repeated measures ANOVAs indicated that there were differences between the means for the support nurses needed to encourage fathers from different backgrounds to attend the visits (pooled *F* (4, 22,897.3) = 20.764, *p* < 0.001). Pairwise t-tests indicated that, compared to Swedish-born fathers, nurses needed relatively more support to encourage fathers with a low-income (*p* < 0.001), foreign-born fathers (*p* < 0.001), and non-Swedish speaking fathers (*p* < 0.001).

**Table 3:** Means, standard errors, and confidence intervals for the additional support nurses need to encourage the attendance of different groups of fathers

	M	SE	95% CI	
			From	To
I need more support to encourage Swedish-born fathers to attend visits	2.78	0.17	2.45	3.11
I need more support to encourage fathers on a low-income to attend visits	3.23	0.18	2.87	3.59
I need more support to encourage foreign-born fathers to attend visits	3.36	0.18	3.00	3.72
I need more support to encourage non-Swedish speaking fathers to attend visits	3.79	0.19	3.41	4.16

## Discussion

The current study examined CHC nurses’ implementation fidelity to a series of new community-based clinical visits for fathers/non-birthing parents during the first year after implementation. The registers showed that of the 80 CHCs providing the three-to-five month visit in 2018, 14 CHCs accounted for over half of all three-to-five month visits, and that 30% of CHCs registered nine or fewer visits during the whole year. Nurses reported the highest adherence for the home visit, while around two-thirds of nurses stated they adhered to the three-to-five month visit. Predictors for adherence varied depending on the home visit, three-to-five week and three-to-five month visit, except when nurses reported receiving enough support to support fathers, which predicted adherence across all three visits. Findings highlighted that nurses reported needing less support for Swedish-born fathers compared to vulnerable fathers, such as those with a low-income, foreign-born, and non-Swedish speaking.

Evaluating implementation fidelity allows for a better understanding of whether the programme was delivered as intended; which helps to inform if the outcomes of the intervention can actually be attributed to the programme [28]. Carroll et al.’s conceptual framework [24] allowed for the assessment of the fidelity of this new clinical visit for fathers to take place in a concise and quantifiable manner via fours aspects: i) coverage, ii) frequency, iii) content and iv) duration.

### Conceptual framework: coverage

Carroll et al. [24], described the coverage of an intervention as its reach; whether every one that is intended to benefit and participate in the programme actually does. Around two-thirds of the nurses in Region Stockholm volunteered to attend the training for the new father/non-birthing parent visits; therefore, fathers who live in locations where nurses did not implement the visits would not be offered them in 2018. It should be noted that CHCs that were both in the pilot scheme and current study were all in the top performing 14 CHCs. This suggests that either pilot locations were more eager to implement the visits and/or that having an extra year to implement the new father visits may yield greater numbers of father visits. Therefore, the amount of fathers/non-birthing parents attending the three-to-five month visit is likely to increase over the coming years. It is important to note however that when it comes to fathers’ attendance at the father visit, it is not known if fathers were not invited or if fathers did not attend their visit despite an invitation. Future research should ask fathers if they were invited, as well as why they did or did not attend, to better understand their motivations and barriers for attending these visits.

### Conceptual framework: frequency

In the current intervention, the frequency is one three-to-five month visit per father. Therefore, to assess the frequency is to examine if the father attended or not. If the average nurse sees 69 infants per year [29], but the average nurse only reported 13.9 visits per year, then, many fathers did not attend the visits, and therefore could not receive any benefits from attending. One reason for this might be that despite nurses’ changing attitudes towards fathers as equal parents [16], some nurses can subconsciously exclude fathers due to opposing values [30]. However, many fathers may also have not attended because they did not believe there was a benefit to attending. Fathers are typically working when the infant is three-to-five months old, and mothers are on parental leave; therefore, some fathers might face work and travel related obstacles to attending [11]. In addition, fathers may be less likely to identify themselves as being depressed and so may find it difficult to seek help [31, 32].

### Concept framework: content

Previous research highlights that community-based interventions should create a measure of fidelity specific to the intervention [33]. The survey items followed the outline in the nurses’ professional guidance [34] to create the overall adherence score for each of the three visits. Nurses adhered to the three-to-five month visit protocol more if they worked at a private CHC, saw more fathers, felt comfortable meeting and supporting fathers, and if they felt they had received enough support, to support fathers. Previous qualitative research from the CHCs who saw the most fathers echoed these findings, stating that believing fathers are worthy of support, actively inviting fathers and physically seeing more fathers helped facilitate adherence to implementing the father visits [30]. They further noted that whenever a new employee started, they would request additional mentoring support so that the new employee would feel confident in implementing the three-to-five month visit [30]. Regarding adherence, seeing more fathers implied that there was a learning process, where nurses’ confidence and competence increased as they saw more fathers. The current study highlighted that after seeing eight or more fathers, nurses started to have higher self-assessed adherence scores. Therefore, child health managers should encourage their nurses to continue seeing fathers and in doing so, they will start to provide higher quality support to fathers.

In the pilot project, nurses highlighted that they felt there were challenges in establishing the new father/non-birthing parent visits and therefore required additional support [14]. The current study showed that receiving enough support and seeing more fathers significantly predicted higher overall self-reported adherence to the three-to-five month visit. Furthermore, nurses stated that they needed more support for fathers on a low-income, that were foreign-born and non-Swedish speaking compared to Swedish-born fathers. If nurses felt they required additional support for these groups of fathers, it was possible that these groups of fathers were less likely to be invited compared to Swedish-born fathers; thus creating greater equity issues. Previous studies show that infants of fathers from ethnic minority communities are more likely to suffer from poor health and risky behaviours later in life [35]. These groups of fathers need to be supported, as involving them can positively affect their child’s lives [35, 36]. It is imperative nurses receive more support, allowing them to feel more capable and confident in supporting potentially vulnerable fathers. Future research should explore the ways additional support can be delivered to nurses and whether minority fathers are invited to a lesser degree compared to Swedish-born fathers.

### Conceptual framework: duration

The duration of the visits, a designated thirty minute visit, was not measured in this study. However, even if nurses self-reported the duration of visits, the self-report would not be able to confirm if a shorter or longer visit was appropriate for that father. Therefore, observational studies are further needed to better understand the duration of the visits. Observations could also occur to further assess the nurses’ implementation fidelity and be compared with their self-reports. Assessing this would be a form of quality control of the service.

## Strengths and limitations

This is the first study to quantitatively assess the implementation of the new clinical visits for fathers/non-birthing parents in Sweden. Within Region Stockholm, the current data represent 69% of all CHCs who saw one or more fathers in 2018. However, how CHC nurses implemented the visits in Region Stockholm may not generalise to other regions, as the trainings and implementation procedures varied by region. In addition, the current study focused on those nurses who volunteered in 2017 to deliver the new clinical father/non-birthing parent visits. In 2019, these visits became a mandatory part of the Swedish child health programme [37], which also included routinely screening fathers for postpartum depression. Therefore, while the current results helped shed light on predictors of adherence during the first year of voluntary implementation, further research is necessary to understand how all nurses implemented these visits.

Although self-reported methods are common in adherence literature, they are inherently subjective and may have limited validity in capturing adherence [33], as nurses may report higher adherence scores based on social desirability [38]. Future research should therefore explore the concurrent perspectives of fathers regarding the nurses’ self-reported adherence, as well as consider using observational methods for measuring adherence.

Another potential limitation is that the number of fathers attending the three-to-five month visit was only obtained on a CHC level. Although, CHCs as a unit decided to implement the visit, it was not known how many fathers were seen by individual nurses. Therefore, it was possible that there might have been an unequal spread of visits per nurse, while the current analysis treated them as seeing an equal number of fathers. However, previous qualitative studies state that nurses within a CHC often work together and support each other as a team [30]; therefore, it is likely that if a CHC sees many fathers, all nurses at that CHC would invite and see fathers.

The generalisability of the findings may be limited because of a potential self-selection of study participants, who may not have been representative of the population of CHC nurses in Region Stockholm. However, in the current nurse sample, 31% of the data came from nurses in the top 14 CHCs, and 17 of the 24 CHCs who saw 1–9 fathers had nurses’ data represented. Therefore, while there is a potential risk of bias due to self-selection, the current study did have a range of participating nurses.

## Conclusion

This is the first study to quantitatively assess a series of clinical visits specifically aimed at fathers/non-birthing parents in Sweden. We show that one year after implementation, the majority of nurses self-report that they adhere to each of the three visits, but that around 30% do not adhere to the three-to-five month visit guidelines. Nurses were more likely to adhere to the three-to-five month visit if they worked at a private CHC, had more fathers attend this visit, and if they felt they received enough support to support fathers. While nurses received a half-day training and two one-on-one mentoring sessions, nurses may need additional support to invite more vulnerable fathers, such as those with a low-income, that are foreign born and non-Swedish speaking compared to Swedish born fathers to attend the visits. Researchers and child health care practitioners can use this information to inform the evaluation of similar interventions in other child health organisations across diverse settings.

## Supplementary Information

**Additional file 1:** **Supplementary table. **List of all item variables regarding nurses’ adherence to each of the three visits (home visit, 3-5 week visit, and 3-5 month visit, respectively).

## Data Availability

The datasets used and/or analysed during the current study are available from the corresponding author on reasonable request.
