# Supplementary material for: The Swedish father/non-birthing parent visit: evaluating implementation fidelity among child health nurses one year after voluntary implementation
Source: BMC Nurs. 2022 Aug 16;21:228. doi: 10.1186/s12912-022-01011-z (PMC9380369; doi:10.1186/s12912-022-01011-z)
Supplement: Supplementary file 1 — Additional file 1: Supplementary table. List of all item variables regarding nurses’ adherence to each of the three visits (home visit, 3-5 week visit, and 3-5 month visit, respectively). [file 12912_2022_1011_MOESM1_ESM.docx]

| **Supplementary table.** List of all item variables regarding nurses’ adherence to each of the three visits (home visit, 3-5 week visit, and 3-5 month visit, respectively). | | |
| --- | --- | --- |
| **Scale** | **Statements** | **% Adhered to Each Item** |
| Adherence to the guidelines for the home visit | I usually invite the father to attend the home visit by asking him | 69 |
|  | I usually introduce myself to the father at home visits in a professional manner | 99 |
|  | It is easy for me to maintain eye contact with the father during home visits | 99 |
|  | I always ask the father about his experience of childbirth | 87 |
|  | I always ask the father how he and his partner divide the parental responsibility between each other | 56 |
|  | I always ask the father if he talks with his partner about how they want to raise their children | 26 |
|  | I always answer the father's questions about parenting in a supportive and helpful manner | 97 |
| Adherence to the guidelines for the 3-5-week visit | I always involve the father when booking the 3-5-week visit | 52 |
|  | I always ask the father when I invite him for a 3-5-week visit | 54 |
|  | I usually introduce myself to the father at the 3-5 week visit in a professional manner | 96 |
|  | It is easy for me to maintain eye contact with the father during the 3-5-week visits | 96 |
|  | I always ask the father how it works for him combining work life or school‎/academic life with family life | 68 |
|  | I always ask the father how he and his partner divide the parental responsibility between each other | 52 |
|  | I always ask the father if he has begun to 'get to know' his child | 82 |
|  | I always ask the father if he is worried about something that concerns his child | 83 |
|  | I always ask the father if he is worried about his parenting skills‎/ability | 74 |
|  | It is easy for me to answer the father's questions about being a parent in a supportive and helpful manner | 96 |
| Adherence to the guidelines for the 3-5-months visit | I always involve the father when booking the 3-5 month visit | 66 |
|  | I always ask the father when I invite him for a 3-5 month visit | 66 |
|  | I always ask the father about his experience regarding parenting | 82 |
|  | I always ask the father if he has made any lifestyle changes following his child's birth (e.g. food, alcohol and tobacco habits, physical activity) | 42 |
|  | I always ask about the father's feelings about how he experiences parenthood (e.g. fear, joy) | 80 |
|  | I always ask the father about how the every day routines with the child are going | 86 |
|  | I always ask the father how he plans his parental leave | 79 |
|  | I always ask the father how he feels (e.g. worry, stress, lifestyle such as tobacco and alcohol habits and recreational drugs) | 79 |
|  | I always ask the father about his sleep habits (e.g. if he is getting enough sleep) | 59 |
|  | I always ask the father if he has space for his own leisure activities | 56 |
|  | I always ask the father how joint parenting is going with his partner | 76 |
|  | I always ask the father about his social network in regards to parenthood (e.g. if they have close relatives that can get support and advice from) | 79 |
|  | I always ask the father if he has any questions about his parenting role | 91 |
